# Detection of Sentinel Lymph Nodes Using Indocyanine Green After Failing Scintigraphy in Merkel Cell Carcinoma

**DOI:** 10.7759/cureus.38453

**Published:** 2023-05-02

**Authors:** Timothy Smith, Francisco A Ferri, Joel Frieder, Lisandro Montorfano, Michael Medina

**Affiliations:** 1 Urology, Detroit Medical Center, Detroit, USA; 2 Plastic and Reconstructive Surgery, Cleveland Clinic Florida, Weston, USA; 3 Surgery, Cleveland Clinic Florida, Weston, USA; 4 Plastic Surgery, Vanderbilt University Medical Center, Nashville, USA; 5 Head and Neck Surgery, Cleveland Clinic Florida, Weston, USA

**Keywords:** merkel cell carcinoma, technetium scintigraphy, new technologies, ­skin cancer, indocyanine green (icg)

## Abstract

Merkel cell carcinoma (MCC) is a rare but highly aggressive skin cancer that carries a high rate of lymph node involvement and death. The National Comprehensive Cancer Network recommends sentinel lymph node (SLN) biopsy for the staging of the disease. Scintigraphy using radioactive isotopes (RI) such as technetium 99m (Tc99) remains the gold standard for the detection of SLNs, however, recently indocyanine green (ICG) fluorescence imaging has been used to aid in the detection of SLNs.We present the case of a patient who presented with MCC of the face and two SLNs successfully identified with ICG fluorescence despite the fact that they were not detected by intraoperative scintigraphy using Tc99. The use of ICG fluorescence imaging in MCC is safe and improves the ability to detect SLNs when combined with RI.

## Introduction

Merkel cell carcinoma (MCC) is a rare but highly aggressive skin cancer that carries a high rate of lymph node involvement and death [1]. In recent years, its incidence in the United States has been increasing. In 2000, its incidence was 0.5 cases per 100,000 population, which grew to 0.7 cases per 100,000 population in 2013 and is expected to continue to climb due to the aging "baby boomer" generation [2].

The principle of sentinel node research in oncology is to be able to highlight and characterize possible dissemination into subclinical lymphatics after locating the first lymph node in a clinical and radiological node-negative tumor. This technique was initially developed by Morton in 1992 in the management of melanoma [3]. The marker initially used was Blue Patent. The development of colloid labeled with technetium 99m (Tc99) allowed for a notable improvement in the detection sensitivity of sentinel lymph nodes (SLNs). SLN status has thus become a major prognostic factor in the management of many malignancies [3].

Similar to melanoma, the National Comprehensive Cancer Network recommends sentinel lymph node biopsy (SLNB) for staging Merkel cell carcinoma [4]. Scintigraphy using radioactive isotopes (RI), such as Tc99, remains the gold standard for the detection of sentinel lymph nodes, however, recently, indocyanine green (ICG) fluorescence imaging has been used to aid in the detection of SLNs [5].

We present a case of a patient who presented with MCC of the face and successful identification of two SLNs with ICG fluorescence, despite the fact that they were both not detected by intraoperative scintigraphy using Tc99.

## Case presentation

**Figure 1:**
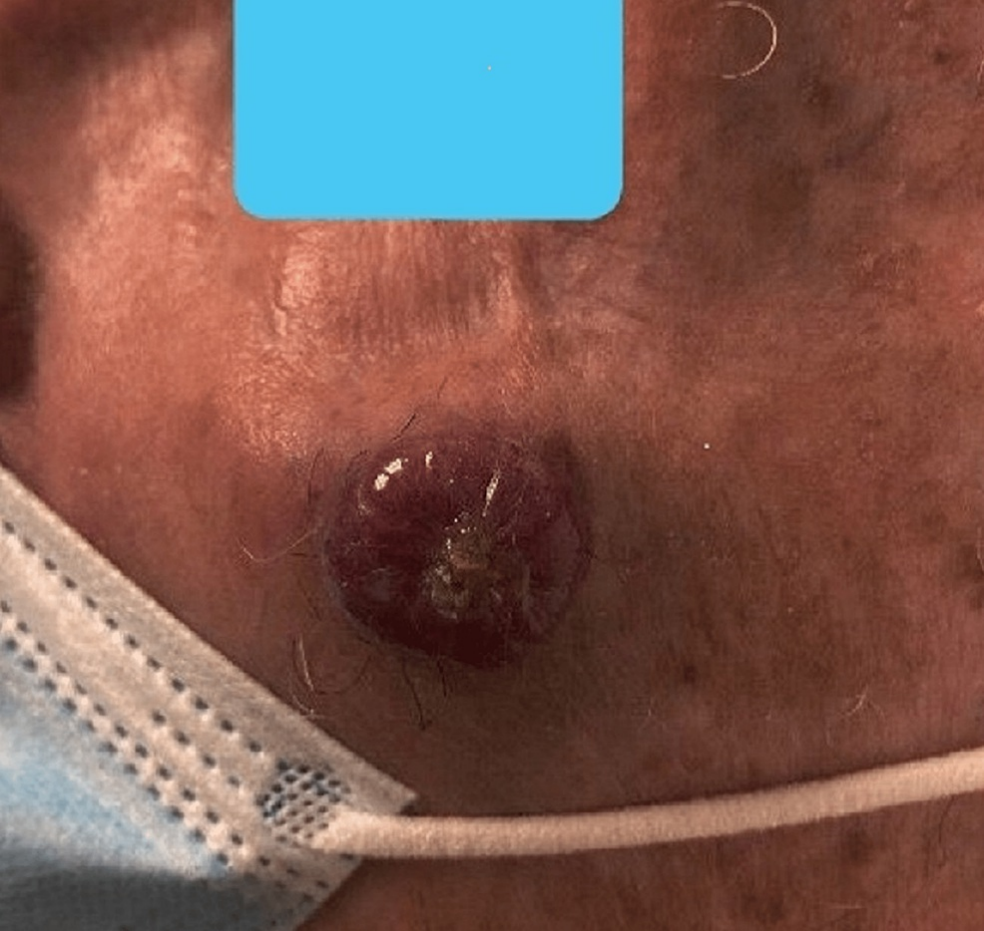
Exophytic lesion in left zygomatic region consistent with Merkel cell carcinoma.

An 85-year-old male presented with a new onset exophytic skin lesion in the left zygomatic region of his head, approximately 2 cm in diameter. The lesion appeared three weeks prior and had been steadily growing (Figure 1). He reported tenderness in the area and denied constitutional symptoms as well as previous treatments. He has a history of actinic keratosis and melanoma in situ on the nose, removed three years prior. The patient acknowledged work-related sun exposure decades ago but not recently.

**Figure 2:**
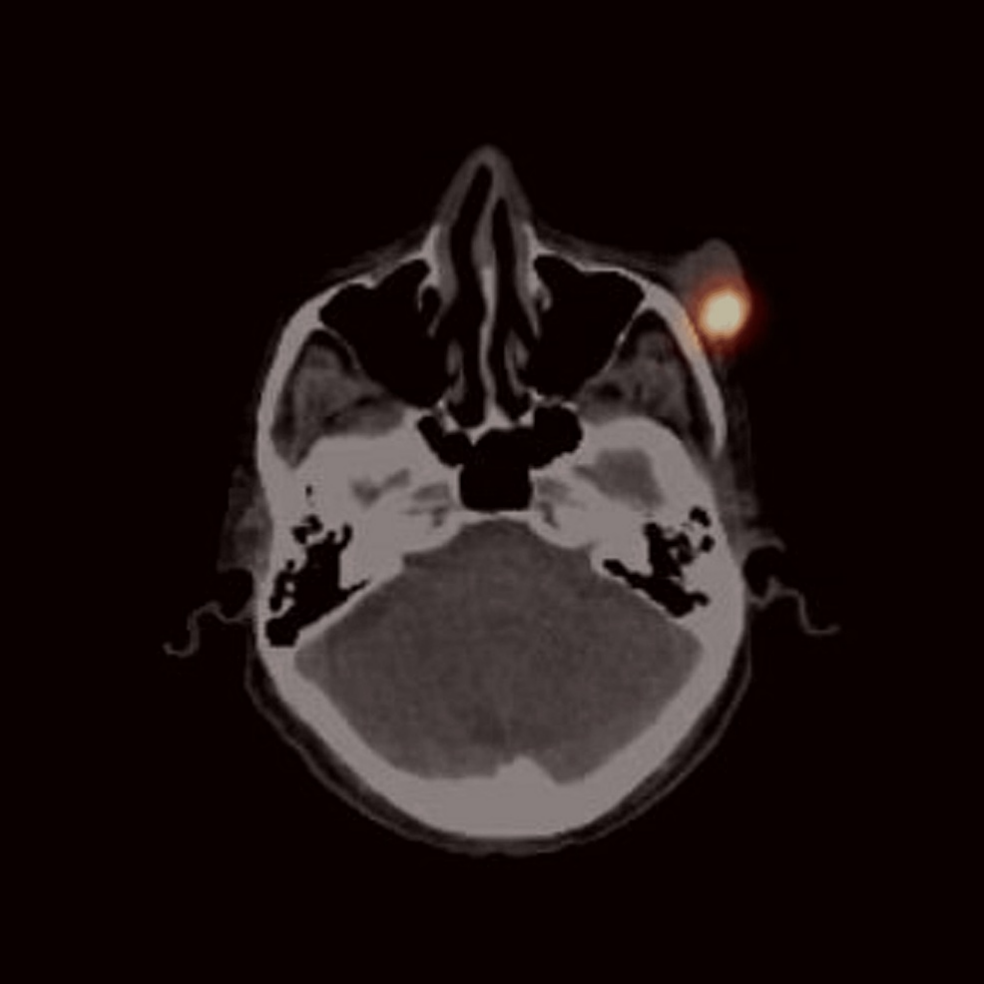
Axial image of fused/SPECT with intralesional injection of Tc99. There were no radiographic foci outside of the lesion that would indicate a sentinel lymph node. SPECT: single-photon emission computerized tomography; Tc99: technetium-99

He was referred to a dermatologist who performed a shave biopsy significant for malignant cells with morphological and immunohistochemical features consistent with MCC. There was no palpable lymphadenopathy. Positron emission tomography-computed tomography (PET-CT) was negative for any signs of metastatic disease. The patient was scheduled for surgical excision and sentinel lymph node biopsy. On the morning of surgery, he had a fused single-photon emission computed tomography (SPECT) with intralesional injection of Tc99, which found no radiographic foci outside of the lesion that would indicate a sentinel lymph node (Figure 2).

**Figure 3:**
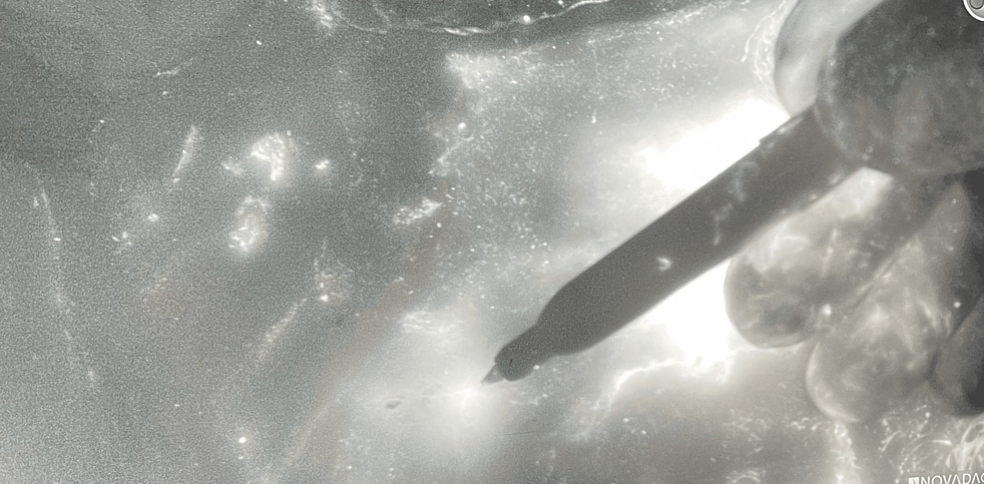
ICG fluorescence using SPY-PHI near-infrared imaging (Kalamazoo MI: Stryker Corporation) showed two fluorescent facial nodes on the left inferior facial region. ICG: indocyanine green

During the operation, a vial of 25 mg of ICG was reconstituted using 10 mL of sterile water and 0.5 mL (1.25 mg) was injected into four quadrants on the periphery of the lesion. The lesion was then surgically excised with margins of 1 cm taken with the specimen. Intraoperative scintigraphy using a hand-held gamma probe was negative for localized foci outside of the primary lesion. ICG fluorescence using SPY-PHI near-infrared imaging (Kalamazoo, MI: Stryker Corporation) showed two fluorescent facial nodes on the left inferior facial region (Figure 3).

The nodes were then excised and scanned again with a gamma probe, which continued to be non-detecting. A review of the SPECT showed that the area where the fluorescent nodes were found was obscured by the signal from the primary injection site. The postoperative course was unremarkable. Final pathology reported a 3.5×3.1×1.8 cm MCC with negative margins. Both lymph nodes were positive for metastatic disease. The patient was then referred to oncology for further treatment of his disease.

## Discussion

Due to its ability to form complexes with albumin, ICG is ideal for evaluating vascular and lymphatic systems. Unlike blue dyes (BDs), ICG fluoresces through the skin, enabling real-time visualization of lymphatic flow and allowing for a more selective dissection and less lymphatic disruption than with RI alone [6]. ICG also has a lower side effect rate (0.01%) when compared to 1.1% with BDs such as isosulfan blue [7].

Establishing SLN status plays a critical role in evaluating the treatment and prognosis of MCC, with five-year survival being 69% for those with lymph node-negative localized disease at the time of presentation vs 39% and 18% for those with lymph node-positive and metastatic disease, respectively [8].

The use of ICG in SLNB for skin malignancies was studied by Fujisawa et al. [9]. In their prospective cohort study, they compared the use of ICG, BDs, and RIs in the detection of SLNs in 34 patients. They found that ICG identified more SLNs per case (p<0.01), more SLNs per basin (p<0.05), and more basins per case (p<0.001) than either RI or blue dye alone. The clinical implications of this study are limited due to its small sample size, however, they were able to show increased efficacy with the use of ICG vs other methods.

The adjunctive use of ICG along with RI in MCC was studied by Knackstedt et al. [10]. In their prospective cohort study of 24 patients, they compared the results of ICG along with RI vs BDs with RI. They found that ICG fluorescence had a higher rate of node localization (89.6%) when compared to BDs (63.3%). Comparable to our case, they also reported one patient for whom an SLN was detected by ICG only and not by RI.

Similarly, a randomized clinical trial analyzing the data from 40 clinically lymph node-negative patients with high-risk head and neck cancers, including MCC, revealed a 100% SLN intraoperative detection rate using ICG with Tc99 vs a 95% detection rate using Tc99 alone (p=0.487) [11]. The authors concluded that the combination of RI and ICG is an attractive option for improving the SLN detection rate in patients with cutaneous head and neck malignancies [11]. Likewise, Nakamura et al. found that the number of SLNs identified by the combination of ICG and RI was greater than that of SLNs identified by RI alone [12].

In our experience, the use of ICG combined with RI brings excellent results in identifying SLNs in head and neck tumors. Interference from the background tissue has not been a problem since there is a noticeable difference between the degree of fluorescence of the SLN compared to the background. In our practice, ICG and RI are used routinely in all patients requiring SLNB for head and neck tumors.

Caution should be taken when deciding to use ICG alone. De Carvalho et al. compared RI, ICG, and BDs independently and found that RI alone was the only method of SLN detection that could stand alone with an SLN detection rate of 92.6% compared to ICG (70.2%), BDs (76.9%), and all three together (95.95%) [13]. The implications of this data are limited, however, the authors state that they were unable to adequately evaluate lesions of the head and neck. It does, however, suggest that there is better efficacy in the use of ICG in combination with RI rather than alone.

Our study has the limitation of being a report of a single case. Further prospective studies with a larger sample size are warranted.

## Conclusions

The use of ICG fluorescence imaging in MCC is safe and improves the ability to detect SLN when combined with RI. ICG fluorescence imaging is relatively inexpensive if the institution already has a near-infrared imaging system being used for other indications. Our case is consistent with the growing body of literature that shows the superiority of the use of ICG in combination with RI for SLNB rather than RI alone or with BDs and has the potential to detect SLNs that would otherwise go undetected.
